# Dr. George E. Hawes

**Published:** 1874-12

**Authors:** 


### OBITUARY.

Dr. George E Hawes.—A meeting of dentists, called at a few hours notice on Tuesday afternoon, (on receipt of the news of the death of Dr. George E. Hawes, of New York City, at Wrentham, Mass.,) was held at the residence of J. G. Ambler, No. 25 West 23d Street, N. Y., August 25th, who presented the following preamble and resolutions, which were unamimously adopted.

*Whereas*, An all-wise Providence has seen fit to remove by death our much esteemed friend and professional brother Dr. George E. Hawes, of this city, therefore

*Resolved*, That while we bow in humble submission to the decrees of Him who doeth all things well, we cannot refrain from expressing our feelings of sadness and sorrow at the loss our profession has sustained in the demise of our much esteemed friend and brother.

*Resolved*, That we show our affections and appreciation for his many virtues and bright examples by placing on record these expressions of our bereavement and sorrow.

*Resolved, That our sympathies true and heartfelt*, are hereby tendered to the family and friends of the deceased, as well as the profession at large in this dispensation of Providence. Though we lament his death, we cannot be unconscious that our loss is his gain.

*Resolved*, That a committee be appointed by the chair to prepare a suitable memorial for publication in the dental and medical journals.

*Resolved*, That these proceedings be published in the *New York Times* and the *Observer*, also a copy sent to the family of deceased.

(Signed,) JOHN ALLEN, *Chairman*.

J. G. AMBLER, *Secretary*.

*Commiitee on Memorial.*—J. G. Ambler, John Allen, W. H. Atkinson, J. Parmly, L. Coville.

### MEMORIAL OF DR. GEORGE E. HAWES.

Dr. George E. Hawes, the subject of this brief memorial, was born at Wrentham, Massachusetts, May 28th, 1810. His Father, Col. George Hawes, was a man of integrity and uprightness; whose patriotism was of an active character in our revolutionary struggles, he filling his place in the ranks of the Colonial patriots, battling against aggressive and oppressive acts of the Mother country, and subsequently serving the State in its Halls of Legislation. His mother still survives him at the advanced age of ninety-five years; having laid all the members of her immediate family in the resting place of the dead. Dr. Hawes commenced his preparatory course as a Dentist in the office of the late Dr. John Lovejoy, and after completing his course of study with him, he commenced the practice of his profession in Park Place, and after years of diligent and successful practice, he located in Bond Street, where he continued his professional career until laid aside by impaired health.

He was an eminently successful Dentist, retaining the confidence of his patients by the perfection of his operations, and his uniform urbanity. By the members of the Dental profession, he was universally esteemed, his opinions on difficult cases in practice were appreciated because they were judicious and sound, and there are none who knew him that do not feel that a safe operator has been lost by the community, and an appreciated and safe counsellor by the profession. We shall miss his acquaintance and counsel. Dr. Hawes, was a *Christian Gentleman*, and to appreciate him, it was only necessary to have his acquaintance. The family who survive him, have, in the recollection of the past, and in the assurance of his blessed future, all the consolation necessary to assuage their grief and make supportable their great bereavement.

(Signed,) J. G. AMBLER,

*On behalf of Committee.*

